# Integration of shot-gun proteomics and bioinformatics analysis to explore plant hormone responses

**DOI:** 10.1186/1471-2105-13-S15-S8

**Published:** 2012-09-11

**Authors:** Yixiang Zhang, Sanmin Liu, Susie Y Dai, Joshua S Yuan

**Affiliations:** 1 Department of Plant Pathology and Microbiology, Texas A&M University, College Station, TX 77843, USA; 2 Institute for Plant Genomics and Biotechnology, Texas A&M University, College Station, TX 77843, USA; 3 Office of Texas State Chemist, Texas A&M University, College Station, TX 77843, USA; 4 Department of Veterinary Pathobiology, Texas A&M University, College Station, TX 77843, USA

## Abstract

**Background:**

Multidimensional protein identification technology (MudPIT)-based shot-gun proteomics has been proven to be an effective platform for functional proteomics. In particular, the various sample preparation methods and bioinformatics tools can be integrated to improve the proteomics platform for applications like target organelle proteomics. We have recently integrated a rapid sample preparation method and bioinformatics classification system for comparative analysis of plant responses to two plant hormones, zeatin and brassinosteroid (BR). These hormones belong to two distinct classes of plant growth regulators, yet both can promote cell elongation and growth. An understanding of the differences and the cross-talk between the two types of hormone responses will allow us to better understand the molecular mechanisms and to identify new candidate genes for plant engineering.

**Results:**

As compared to traditional organelle proteomics, the organelle-enrichment method both simplifies the sample preparation and increases the number of proteins identified in the targeted organelle as well as the entire sample. Both zeatin and BR induce dramatic changes in signaling and metabolism. Their shared-regulated protein components indicate that both hormones may down-regulate some key components in auxin responses. However, they have shown distinct induction and suppression of metabolic pathways in mitochondria and chloroplast. For zeatin, the metabolic pathways in sucrose and starch biosynthesis and utilization were significantly changed, yet the lipid biosynthesis remained unchanged. For BR, lipid biosynthesis and β-oxidation were both down-regulated, yet the changes in sucrose and starch metabolism were minor.

**Conclusions:**

We present a rapid sample preparation method and bioinformatics classification for effective proteomics analysis of plant hormone responses. The study highlighted the largely differing response to zeatin and brassinosteroid by the metabolic pathways in chloroplast and mitochondria.

## Background

### Exploring the plant proteome

Proteomics can directly address many biological questions by revealing the abundance of certain proteins within organisms. Traditionally, two-dimensional polyacrylamide gel electrophoresis (2D-PAGE) was the golden standard for proteomics analysis, yet the platform is limited by both protein identification and quantification capacities. The recent advances in mass spectrometry instrumentation, separation methods, data acquisition and analysis tools have enabled use of the so-called 'shot-gun' proteomics. It uses tandem mass spectrometry and the multidimensional protein identification technology (MudPIT) [1]. In the MudPIT platform, the whole proteome is directly digested with protease, and the resulting peptides are subjected to multidimensional chromatography separation. The separated peptides are then analyzed online by mass spectrometry. The so called MudPIT platform eliminates the tedious gel separation and has been broadly applied in plant biology studies [2,3]. Even though the platform has superior performance as compared to 2-D gel platforms, limitations still exist for several reasons. First, profiling the whole proteome is complicated by the complexity of the protein sample, the number of proteins expressed, the differing molecular weights, and other variations in chemical and physical characteristics[4,5]. Also, many functional proteins such as GTPases, kinases and phosphatases exist in low abundance. Their signals can be easily masked by highly abundant proteins such as ribulose 1, 5-bisphosphate carboxylase/oxygenase (Rubisco) [6]. These challenges can be addressed by improving sample preparation methods, bioinformatics analysis, sample processing, and mass spectrometry instrumentation. We hereby present the integration of a rapid sample preparation method with bioinformatics analysis to achieve better peptide identification and focused study of chloroplast and mitochondrial proteins.

### Proteomics for plant organelle

We are particularly interested in chloroplast and mitochondria because the two organelles are important for energy metabolism and plant growth, among many other functions. In particular, the proteome dynamics of these two organelles in response to growth relevant hormones like auxin, cytokine, and brassinosteroid will shed light onto the mechanisms for plant hormone responses. It will also identify candidate genes for improving crop seed and biomass yield for food, fiber and energy usages.

Traditionally, in order to identify proteins in a particular type of organelle, the organelle is separated by gradient density centrifugation and ultra-centrifuged from a large quantity of initial samples [6,7]. Proteomics studies toward specific organelles have been done on nuclei, mitochondria, chloroplasts, Golgi apparatuses, and endoplasmic reticulum, etc. [5,8]. For example, Dunkley and colleagues used localization of organelle proteins by isotope tagging (LOPIT) to simultaneously localize 527 proteins out of 689 proteins identified in several organelles of Arabidopsis [9]. Most of the traditional organelle purification involves time-consuming and tedious separation steps, which could introduce extra errors [10]. We hereby simplified the traditional method of organelle separation by implementing a rapid centrifugation step. The rapid sample preparation method integrated with bioinformatics classification was evaluated as an alternative to study mitochondria and chloroplast proteomics in plant's responses to growth hormones.

### Proteomics comparison of plant response to zeatin and brassinosteroid

The proteomics analysis of hormone responses is part of our long-term efforts to identify important genes involved in plant biomass increases for bioenergy purposes. Several plant hormones such as auxin and gibberellic acid can promote plant growth and are known to be able to increase plant biomass accumulation through different mechanisms. Among these plant hormones, zeatin and BR are of particular interest to us. Zeatin is a plant hormone belonging to cytokinins and regulates plant development and growth. Zeatin has been widely applied in agriculture to increase fruit or seed size and is well known to promote cell elongation and root development [11]. Interestingly, BR is also known to be able to promote plant growth through cell elongation [12]. Even though both plant hormones can promote cell elongation and growth, the underlying mechanisms are believed to be widely different; the hormone signaling pathways for the two are unique to one another. However, very few studies have focused on studying the differences and cross-talk between the responses in the two hormones at the proteome level. We hereby utilized the aforementioned platform to explore the proteome responses of Arabidopsis in response to treatment by the two plant hormones. The metabolic pathways in chloroplasts and mitochondria are of the particular interest.

Overall, in this article, we have integrated a simple sample preparation method with bioinformatics classification to analyze plant responses to zeatin and BR. The new method has been shown to improve protein identification, in particular in mitochondria. Using this platform, we have revealed that both zeatin and BR induce significant changes in signaling and metabolism. The shared regulated protein components indicated that both hormones may down-regulate some components in auxin responses. However, the two plant hormones have shown distinctive induction and suppression of metabolic pathways. For zeatin, the metabolic pathways in sucrose and starch biosynthesis and utilization were significantly up-regulated, yet the lipid biosynthesis remained unchanged. For BR, the lipid biosynthesis and β-oxidation were both down-regulated, yet the changes in sucrose and starch metabolism are minor. These differences highlight the different molecular and metabolic mechanisms for response to zeatin and BR. The data can help us to design better strategies to promote plant biomass accumulations.

## Methods

### Plant material and growth conditions

*Arabidopsis thaliana *ecotype Col-0 was used. Seeds were stratified at 4°C to synchronize germination for 2 days and then grown at 23°C/19°C under a 12 h/12 h light/dark cycle for 4 weeks.

### Hormone treatment

Zeatin (Sigma-Aldrich, St. Louis, MO) and 24-epibrassinolide (PhytoTechnology Laboratories, Shawnee Mission, KS) were sprayed at 100 μm and 0.5 mg/L, respectively. 0.8% methanol solution was sprayed as mock. The aerial parts of plant were collected at 24 hours after the spray.

### Plant total protein isolation

For the total protein isolation, a plant total protein extraction kit (Sigma-Aldrich, St.Louis, MO) was used, and the entire procedure followed the manufacturer's manual. 150 mg of aerial tissue from Arabidopsis was collected and ground in liquid nitrogen to a fine powder. Pre-cooled methanol solution with protease inhibitor was added to the powder and vortexed for 30 seconds. The mixture was incubated at -20°C then centrifuged at 16,000 × g for 5 minutes at 4°C. Supernatant was removed and the pellet was washed by methanol solution for two more times. The resulting pellet was washed by pre-cooled acetone and centrifuged at 16,000 × g for 5 minutes at 4°C. SpeedVac was used to remove residual acetone and Reagent Type 4 Working Solution provided by the kit was used to incubate the pellet for 15 minutes at room temperature. The pellet was then centrifuged at 16,000 × g for 30 minutes, and supernatant was collected and stored at -80°C for future proteomics use (See Additional File 1).

### Organelle enrichment and protein isolation

The organelle enrichment procedure was developed based on the method from Santoni [13] with some modification. 5 g of fresh aerial tissue of Arabidopsis was collected and washed by ice-cold water to remove the soil. A blender was used to disrupt the tissue after adding a 2:1 (mL medium/g fresh weight) homogenization buffer (50 mM TRIZMA base, 500 mM Sucrose, 10% Glycerol, 20 mM EDTA-Na_2_, 20 mM EGTA, 50 mM NaF, 5 mM beta-glycerophosphate, 1 mM phenantroline, 0.6% PVP40, 10 mM ascorbic acid, 1 mM leupeptin, 5 mM DTT, 1 mM Naorthovanadate, pH 8.0 adjusted by MES). The homogenate was then filtered through Miracloth to remove plant debris. Centrifugation of filtered homogenate was conducted at 1,000 × g for 5 minutes to remove the nuclei. The supernatant was then centrifuged at 26,000 × g for 25 minutes to pellet organelles.

For protein isolation of enriched organelles, pre-cooled methanol with protease inhibitor was added to the organelle-enriched pellet, which was collected after the centrifugation described in the Organelle enrichment section. The sample was then vortexed for 30 seconds. The mixture was incubated at -20°C then centrifuged at 16,000 × g for 5 minutes at 4°C. The supernatant was removed and the pellet was washed twice by methanol solution. The resulting pellet was again washed by pre-cooled acetone and centrifuged at 16,000 × g for 5 minutes at 4°C. Residual acetone was removed by SpeedVac, and Reagent Type 4 Working Solution was used to incubate the pellet for 15 minutes at room temperature. The pellet was then centrifuged at 16,000 × g for 30 minutes, and supernatant was collected and stored at -80°C for future proteomics use (See Additional File 1A).

### MudPIT

MudPIT-based shot-gun proteomics was carried out to analyze each sample. Approximately 100 μg of protein was digested by Trypsin Gold, Mass Spectrometry Grade (Promega, WI, USA) with 1:40 w/w at 37°C for 24 h. The digested peptides were desalted using a Sep-Pak plus C18 column (Waters Limited, ON, Canada) and then loaded onto a biphasic (strong cation exchange/reversed phase) capillary column using a pressure tank. The 2D back column was composed of 5 cm of C18 reverse phase resin and 3 cm of strong cation exchange (SCX) resin. The back column was then connected to a 15-cm-long 100 um-ID C18 column (packed in house with the same C18 reverse phase in the back column) and sprayed through a SilicaTip (New objective, Inc, Woburn, MA). The two-dimensional liquid chromatography separation and tandem mass spectrometry conditions followed the protocols previously described by Washburn et al. [14]. Before SCX separation, a 1 h RP gradient from 100% Solvent A (95% H_2_O, 5% ACN, and 0.1% formic acid) to 100% Solvent B (30% H_2_O, 70% ACN, and 0.1% formic acid) was configured to move peptides from C18 resin to SCX resin in the back column. The SCX LC separation was performed with eleven salt pulses containing increasing concentrations of ammonium acetate. Each salt pulse was followed by a 2 h reverse phase gradient from 100% Solvent A to 60% Solvent B. The LC eluent was directly nanosprayed into a linear ion trap mass spectrometer, Finnigan LTQ (Thermo Fisher Scientific, San Jose, CA). The mass spectrometer was set to the data-dependent data acquisition mode, and full mass spectra were recorded on the peptides over a 300-1700 m/z range, followed by five tandem mass (MS/MS) events for the most abundant ions from the first MS analysis. The Xcalibur data system (Thermo Fisher Scientific, San Jose, CA) was used to control the LC-LTQ system and collect the data.

### Data analysis

Tandem mass spectra were extracted from the raw files and converted into the MS2 file. The MS2 file was searched against the Arabidopsis protein database downloaded from The Arabidopsis Information Resource (TAIR); it contains reverse sequence and common contaminant proteins. A ProLuCID algorithm was used to search for data using the Texas A&M Supercomputing Facility. The validity of peptide/spectrum matches was assessed in DTASelect2.0 using a 0.05 false discovery cutoff, with a cross-correlation score (XCorr) that's larger than 1, and normalized difference in cross-correlation scores (DeltaCN) larger than 0.08. Proteins with more than two peptides were identified as detected and were recorded.

### Ontology and pathway analysis

PatternLab [15] software is used for data analysis to discover differentially expressed proteins. The cutoff of p-value and Fold-change is 0.05 and 2.0 respectively. Gene ontology annotations for proteins were performed by VirtualPlant [16]. The pathway analysis of proteins differentially expressed was analyzed by Aracyc http://www.arabidopsis.org/biocyc/. Cluster analysis was carried out by MeV[17].

### Protein classification software

A python package was developed to parse proteins based on their GO keywords (See Additional File 1B and Additional File 2). The report containing differentially expressed proteins searched each protein ID against GO Slim, which can be downloaded from ftp://ftp.arabidopsis.org/home/tair/Ontologies/Gene_Ontology/. If the annotation of the protein matches the keyword set, the ID will be output to a text file and the number of matched protein ID will be displayed.

## Results

### The organelle enrichment method improved total and mitochondrial protein identification

The protein identification and mass spectra were compared between samples prepared by the organelle enrichment and traditional methods. The average number of proteins identified from the organelle enrichment samples and those from total protein isolation was 3099 and 2897, respectively (shown in Table 1). The average identified peptide increased from 20128 to 21547. The average spectra count increased 23.44%, from 55565 to 68588. The pair-wise student's t-test showed a significant difference for the number of peptides and spectra count between the organelle enriched sample and the traditional protein sample (Table 1). However, there was not a significant difference in the number of identified proteins. This is probably due to the dynamic range of the gel-free shotgun proteomics platform, which determines the detection up-limit of the described platform. The protocol used here presents a digestion of about 100 μg of the total protein, tryptic digestion, and chromatography separation. Prior research with a similar platform also reported similar protein identification numbers of the global proteome profiling and/or similar peptide counts and spectra counts [18,19].

**Table 1:** Improved protein identification using the organelle enrichment method (OEM) as compared traditional method (TM)

	Protein Identified*	Peptide IDs*	Spectra Count*	Mitochondrial proteins	Percentage of mitochondrial proteins (%)
OEM 1	2956	21283	73181	228	7.71
OEM 2	3121	21848	68127	236	7.56
OEM 3	3221	21511	64458	201	6.24
TM sample 1	2880	20386	64733	179	6.22
TM sample 2	2732	19939	52752	170	6.22
TM sample 3	3081	20061	49211	181	5.87
The pair-wise student's t-test	0.0845	0.0295	0.04	<0.01	

*All of the data are filtered and forward matches

We further processed the proteomics data with protein classification software. The analysis indicated the organelle enrichment method has identified over 30% more mitochondrial proteins, even though the chloroplast protein identification didn't change significantly. As compared to the traditional method, the organelle enrichment method led to a greater percentage of mitochondrial protein identified (Table 1). This suggests our protocol has effectively enriched the proteins in mitochondria for the research purpose. Overall, with this simplified sample preparation method, we successfully enriched mitochondrial protein and identified more proteins that are involved in energy metabolism. The integration of bioinformatics classification allowed us to focus more on mitochondrial pathways. We therefore used this method to explore the proteome dynamics during plant hormone responses.

### Overview of zeatin and BR-regulated proteins

**Figure 1:**
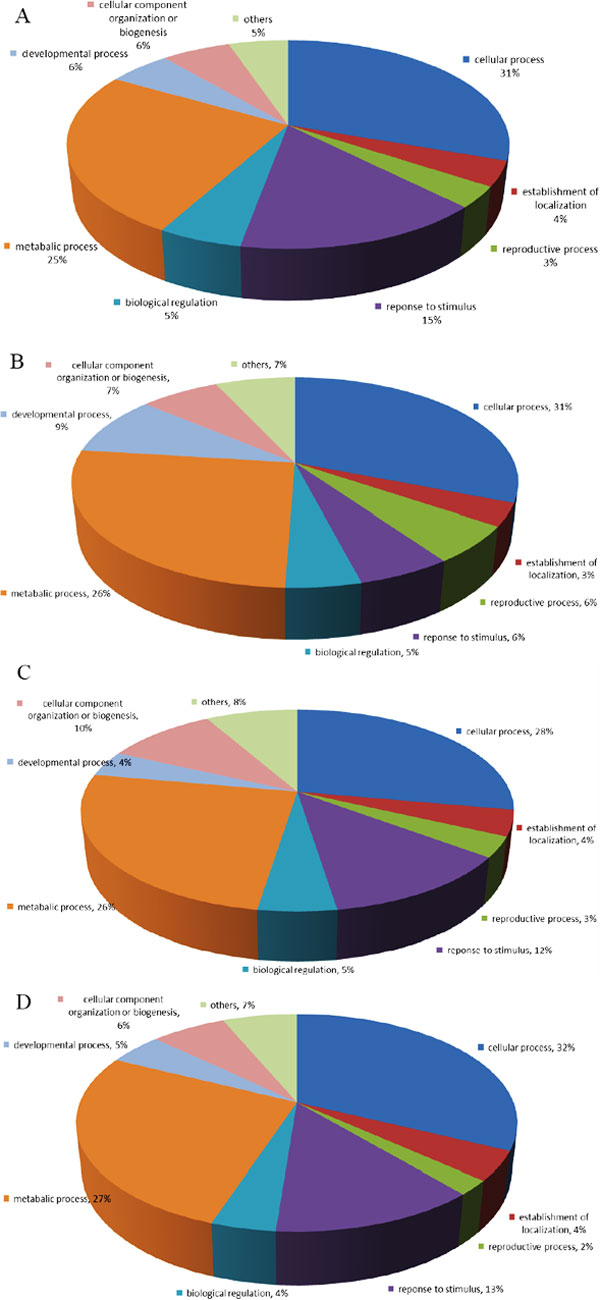
**Pie charts of GO distribution of up-regulated and down-regulated proteins in zeatin (A and C) and BR (B and D) treated Arabidopsis according to their biological process**.

As aforementioned, we focused on comparing zeatin and BR treated *Arabidopsis thaliana *Col-0 plants with wild-type plants. A total of 267 proteins were up-regulated and 88 were down-regulated in the zeatin-treated sample (See Additional File 3). A total of 60 up-regulated and 228 down-regulated proteins were identified in BR treated samples (See Additional File 4). These proteins could be classified into several groups based on the biological process category of GO. As shown in Figure 1A, most zeatin-triggered proteins are involved in cellular processes (36%), compared with metabolic processes (25%), response to stimuli (15%), developmental processes (6%), cellular component organization or biogenesis (6%), biological regulation (5%), etc. Figure 1B shows the category percentage of up-regulated proteins in BR treated plants: cellular processes (31%), metabolic processes (26%), developmental processes (9%), cellular component organization or biogenesis (7%), response to stimuli (6%), biological regulation (5%), etc.

Among the differentially regulated proteins between the two hormone treatments, we particularly focused on the shared genes as shown in Table 2. Among all of the differentially expressed proteins present in both of the zeatin and BR treated samples, a total of 12 proteins were up-regulated in both treatments. These proteins include DEAD/DEAH box helicase (AT3G18600), 5'-adenylylsulfate reductase 2 (APR2, AT1G62180), protoporphyrinogen oxidase (putative, AT5G14220), co-chaperone grpE family protein (AT5G17710), and others. A second group contains the 35 genes that are down-regulated in both samples. This group contains sulfate adenylyltransferase 4 (APS4, AT5G43780), auxin-binding protein 1 (ABP1, AT4G02980), jacalin lectin family protein (AT2G33070), plastid-lipid associated protein (PAP, AT2G46910) and oligosaccharyl transferase STT3 subunit family protein (STT3A, AT5G19690) and others. Only two genes were found with opposite regulation. AT2G40360 was up-regulated in the zeatin-treated sample but down-regulated in BR treated samples. Kiba and colleagues found this gene up-regulated after cytokinin treatment, confirming our studies [20].

**Table 2:** Shared differentially expressed proteins between zeatin and BR treated samples

Gene Locus	Fold change (zeatin)	Fold change (BR)	Description
AT3G53520	15.44	9.79	NAD-dependent epimerase/dehydratase family protein
AT5G17710	9.07	6.72	co-chaperone grpE family protein
AT2G33430	8.45	5.69	plastid developmental protein DAG, putative
AT3G18600	8.36	3.52	DEAD/DEAH box helicase, putative
AT1G26340	7.74	4.54	cytochrome b5, putative
AT5G44320	7.70	3.52	eukaryotic translation initiation factor 3 subunit 7
AT5G08260	6.49	4.09	serine carboxypeptidase S10 family protein
AT1G62180	5.83	3.52	5'-adenylylsulfate reductase 2
AT5G18280	5.83	3.07	apyrase (APY2)
AT5G38990	4.49	3.07	protein kinase family protein
AT5G28050	2.12	2.49	cytidine/deoxycytidylate deaminase family protein
AT5G14220	2.10	2.01	protoporphyrinogen oxidase, putative
**AT2G40360**	2.09	-3.33	transducin family protein/WD-40 repeat family protein
AT1G52410	-2.10	-2.15	caldesmon-related
**AT1G60420**	-2.33	2.05	DC1 domain-containing protein
AT4G00620	-2.33	-2.93	tetrahydrofolate dehydrogenase/cyclohydrolase, putative
AT1G49820	-2.33	-2.93	5-methylthioribose kinase family SEC14 cytosolic factor family
AT1G55690	-2.33	-2.93	protein/phosphoglyceride transfer family protein
AT4G02980	-2.65	-3.33	auxin-binding protein 1 (ABP1)
AT1G66070	-2.65	-3.33	translation initiation factor-related
AT4G16580	-2.65	-3.33	expressed protein
AT5G33320	-2.65	-3.33	triose phosphate/phosphate translocator, putative
AT1G06650	-2.65	-3.33	2-oxoglutarate-dependent dioxygenase, putative
AT4G30840	-2.65	-3.33	WD-40 repeat protein
AT1G05560	-2.65	-3.33	UDP-glucose transferase (UGT75B2)
AT3G14010	-2.65	-3.33	hydroxyproline-rich glycoprotein family protein
AT2G43160	-2.65	-3.33	epsin N-terminal homology (ENTH) domain- containing protein
AT2G32810	-2.65	-3.33	beta-galactosidase, putative/lactase, putative
AT2G38000	-2.97	-3.73	chaperone protein dnaJ-related
AT5G40170	-2.97	-3.73	disease resistance family protein
AT2G34680	-2.97	-3.73	AIR9
AT4G24090	-3.10	-3.91	expressed protein
AT1G16860	-3.10	-3.91	merozoite surface protein-related
AT5G23210	-3.10	-3.91	SCPL34, similar to serine carboxypeptidase S10 family
AT3G56130	-3.29	-4.13	biotin/lipoyl attachment domain-containing protein
AT1G53590	-3.29	-4.13	C2 domain-containing protein
AT2G33070	-3.88	-4.89	jacalin lectin family protein
AT2G28760	-4.06	-5.11	NAD-dependent epimerase/dehydratase family protein
AT4G26555	-4.06	-5.11	immunophilin/FKBP-type peptidyl-prolyl cis- trans isomerase family protein
AT3G63150	-4.06	-5.11	GTP-binding protein-related
AT2G46910	-4.20	-5.29	plastid-lipid associated protein PAP/fibrillin family protein
AT5G19690	-4.84	-6.09	oligosaccharyl transferase STT3 subunit family protein
AT2G33830	-5.61	-7.07	dormancy/auxin associated family protein
AT5G05740	-5.75	-2.41	S2P-like putative metalloprotease
AT5G43780	-6.25	-3.41	sulfate adenylyltransferase 4/ATP-sulfurylase 4 (APS4)
AT1G33360	-7.80	-9.82	ATP-dependent Clp protease ATP-binding subunit ClpX, putative
AT3G53520	-8.90	-11.20	NAD-dependent epimerase/dehydratase family protein
AT4G36530	-10.08	-4.63	hydrolase, alpha/beta fold family protein
AT5G22880	-26.74	-4.23	histone H2B, putative

*IDs in bold are genes with opposite regulation

### Cluster analysis of zeatin and BR treated sample

**Figure 2:**
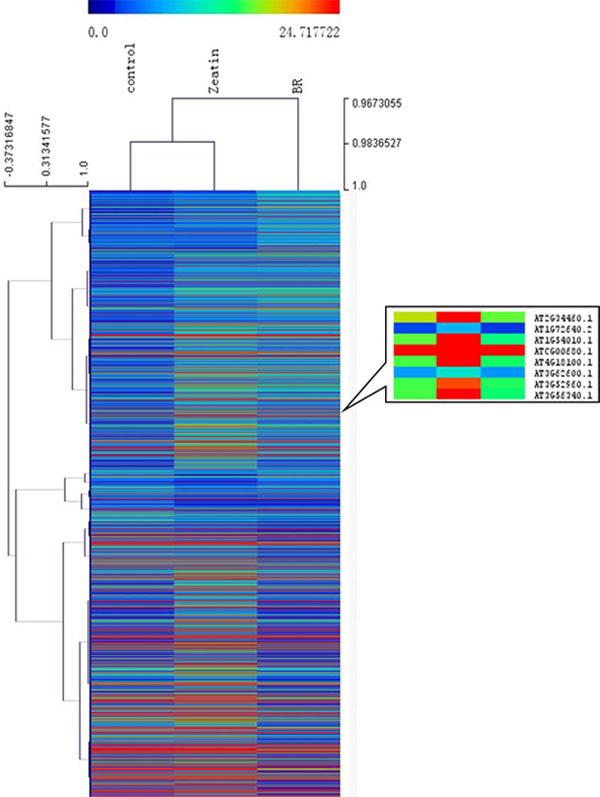
**Overview of cluster analysis of zeatin and BR treated samples and a snapshot of a group of zeatin proteins**.

Besides the differentially regulated genes, we further carried out two types of global analysis, the cluster analysis of protein abundance based on normalized mass spectra counts and the pathway analysis of differentially regulated proteins. Figure 2 shows the overview of cluster analysis; it revealed a dynamic proteome profile among the wild type, the zeatin treated sample, and the BR treated sample. It also revealed that many proteins with similar function showed similar expression patterns. We focused particularly on some mitochondria and chloroplast-located proteins. One group of the zeatin treated, specific up-regulated proteins contains: AT2G34460 (NAD(P)-binding Rossmann-fold superfamily protein), AT1G72640 (NAD(P)-binding Rossmann-fold superfamily protein], AT1G54010 (GDSL-like Lipase/Acylhydrolase superfamily protein), ATCG00680(subunit of the photosystem II reaction center), and others. The first three genes are involved in lipid metabolism, especially lipid oxidation and catabolism.

### Pathway analysis revealed distinctive responses of two hormones

Both zeatin and BR are relevant to plant growth regulation, cell elongation, and energy metabolism. We therefore carried out pathway analysis using AraCyc to investigate if both hormone treatments promote the plant and cellular growth with the same metabolic pathway or not. The pathway analysis revealed distinctive patterns.

**Figure 3:**
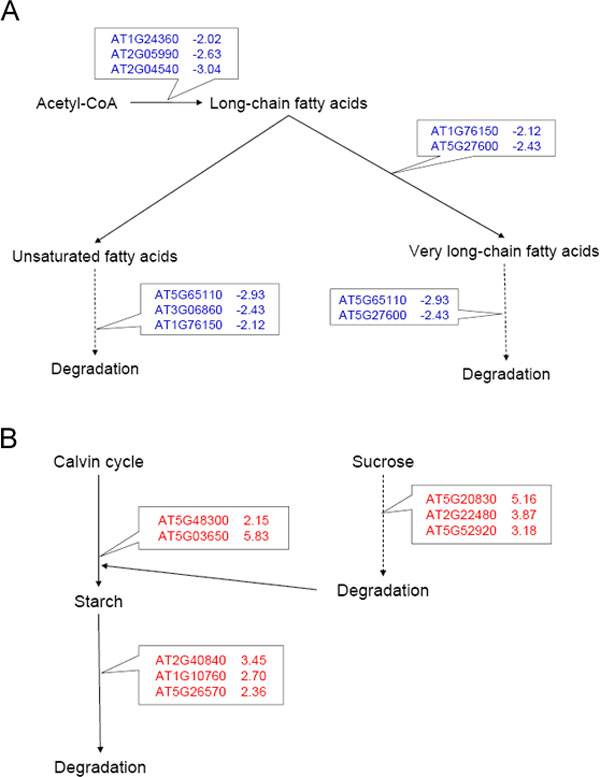
**Pathways analysis of differentially regulated proteins in zeatin and BR responses**. A. BR down-regulates many lipid biosynthesis sand utilization proteins. B. Zeatin up-regulates many sucrose and starch metabolism proteins.

The most impressive pathway-level differences between zeatin and BR triggered responses are the regulation within the fatty acid biosynthesis pathway. Three proteins (AT1G24360, AT2G05990 and AT2G04540) were found down-regulated in the BR treated sample. AT1G24360 is an NAD(P)-binding Rossmann-fold superfamily protein, and AT2G05990 is an enoyl-ACP reductase, a component of the fatty acid synthase complex. AT2G04540 is a beta-ketoacyl synthase. All of these enzymes are involved in fatty acid biosynthesis and elongation. In addition, two other proteins relevant to very long-chain fatty acid biosynthesis were down-regulated. These two proteins are AT1G76150 encoding an enoyl-CoA hydratase and AT5G27600 encoding a peroxisomal long-chain acyl-CoA synthetase. Despite the many down-regulated proteins in BR responses, few proteins can be found differentially expressed in the zeatin treated sample for lipid biosynthesis (Figure 3).

Besides the down-regulation of fatty acid biosynthesis, the pathways for utilization and oxidation of fatty acid were also down-regulated in BR treated samples. Four down-regulated gene products (AT1G76150, AT3G06860, AT5G65110 and AT5G27600) play distinct roles in fatty acid β-oxidation pathway. AT1G76150 degrades even *cis*-unsaturated fatty acids. AT5G65110 encodes an acyl-CoA oxidase for fatty acid oxidation. AT5G27600 involves in oxidation of very long chain fatty acid in peroxisomes.

Even though lipid metabolisms were significantly changed in response to BR treatment, sucrose and starch metabolisms seem to be changed more by zeatin treatments. Two gene products in the starch biosynthesis pathway were up-regulated in the zeatin-treated sample. These two proteins are AT5G48300, a small subunit of ADP-glucose pyrophosphorylase and AT5G03650, starch branching enzyme. Meanwhile, proteins were found up-regulated in both the starch degradation pathway I and pathway II. Among these proteins are AT2G40840, a crucial enzyme for starch to sucrose conversion; AT1G10760, α-glucan dikinase; and AT5G26570, chloroplastidic phosphoglucan water dikinase. However, in the BR treated sample, only ApL4, a large subunit of ADP-glucose pyrophosphorylase catalyzing the first rate limiting step in starch biosynthesis was found down-regulated in the starch biosynthesis pathway.

In addition to starch metabolism, zeatin treatment has induced three enzymes in sucrose biosynthesis and catabolism. These enzymes are AT5G20830 (sucrose synthase, Sus1), AT2G22480 (phosphofructokinase) and AT5G52920 (pyruvate kinase beta subunit). No proteins in the sucrose degradation pathway showed significant changes in the BR treated sample.

## Discussion

### Plant organelle proteomics

Different strategies can be used for proteomics analysis in organelles like mitochondria and chloroplast [21]. The traditional approach is to isolate these organelles with multiple steps of gradient centrifugation and ultra-centrifugation. Proteins were further isolated from the organelles for proteomics analysis. The limitation of the strategy lies in the requirement of a large quantity of initial sample and the potential errors that could be introduced during the multiple step purification [22,23]. We hereby adopted another strategy to combine a simple and rapid sample preparation method with bioinformatics classification. One simple centrifugation step was used to separate the mitochondria and chloroplast from other plant organelles. The separated mitochondria and chloroplast protein was then used for shot-gun proteomics and bioinformatics classification. The method has led to the enrichment of mitochondrial protein identification by 30%, and reduction of initial sample amount by more than 10-fold. We went ahead and utilized the method to study an important biological question in plant hormone responses.

### Improved protein identification for hormone response proteomics analysis

Previous research, mainly utilizing 2D DIGE (Two-dimensional differential gel electrophoresis), was carried out to study the BR-treated Arabidopsis [24]. The study has led to the discovery of 103 of differentially expressed proteins. As compared to the previous studies, more differentially proteins were identified in the presented study, demonstrating the effectiveness of the shot-gun proteomics platform and our sampling strategy. A total of 355 proteins have been identified in the zeatin treated samples and a total of 288 proteins were found to be differentially expressed in the BR treated samples. The deep coverage of differentially regulated proteins and focused study of energy-related pathways in mitochondria and chloroplasts allow us to have a global comparison of metabolic pathway regulations at the proteome level between the two types of hormone responses.

### The distinct and shared pathways induced by zeatin and BR treatment

The study has revealed significantly differential regulation of metabolic pathways in zeatin and BR, in particular for pathways located in mitochondria and chloroplasts. Even though both zeatin and BR can promote cell elongation, the mechanisms are expected to be different. Our results highlighted that BR down-regulates key proteins in both fatty acid biosynthesis and oxidation. Fatty acid β-oxidation eventually breaks down the long-chain fatty acids and produces acetyl-CoA to enter TCA cycle [25]. The fact that both fatty acid biosynthesis and catabolism are down-regulated indicates that BR may promote the cell elongation and growth through shutting down the energy storage through lipid biosynthesis. Interestingly, zeatin treated plants showed essentially no changes in these two pathways, indicating a completely different metabolic regulatory mechanism.

For zeatin treatment, some of the sucrose and starch biosynthesis proteins were up-regulated. Additionally, proteins involved in sucrose and starch degradation were also up-regulated. The use of sucrose is one way that plants transport energy; synthesized sucrose from photosynthetic tissues can be transported to other tissues and cells for utilization [26]. The fact that both biosynthesis and degradation were up-regulated indicates the rapid metabolism of these energy source compounds. Interestingly, BR treatment only induces the down-regulation of one gene involved in starch biosynthesis.

The comparison of the two hormone responses indicated that the two types of plant hormones regulate cell elongation and growth through distinctive pathways. BR down-regulates key proteins in lipid metabolisms and energy storage, while zeatin up-regulates key proteins in sucrose and starch metabolisms for energy utilization. The future work can be developed to coordinate the expression of genes involved in the responses to two plant hormones to develop new ways for manipulating plant growth and development.

## List of abbreviations used

MudPIT: Multidimensional protein identification technology); BR: brassinosteroid; Rubisco: ribulose 1, 5-bisphosphate carboxylase/oxygenase; 2D-PAGE: two-dimensional polyacrylamide gel electrophoresis

## Competing interests

The authors declare that they have no competing interests.

## Authors' contributions

YZ conducted experiments, performed the analysis and drafted the manuscript. SL coded the protein classification package. JSY and SYD participated in the design of the study and helped to draft the manuscript. All authors read and approved the final manuscript.

## Supplementary Material

Additional file 1 **Workflow of sample preparation and bioinformatics analysis**. Additional file 1A shows the workflow of organelle enrichment, protein isolation and 2D LC/MS/MS. Additional file 1B illustrates the flow of protein classification package. Additional file1C shows workflow of traditional plant total protein isolation by TCA/acetone. Click here for file

Additional file 2 **Protein classification software for organelles enrichment analysis**. The package was developed by Python. For usage, open the code in text editor and type the key word in Keywords function. Follow the instruction at the beginning of the package. Click here for file

Additional file 3 **All differentially expressed proteins in zeatin treated sample**. The list was generated by PatternLab. The data from DTASelect were normalized by Row Sigma method and processed by TFold pairwise analysis. The minimum signal in all classes is 2. The cutoff of fold change, p-value and Benjamin-Hochberg (BH) theoretical false-positive rate are 2.0, 0.05 and 0.1, respectively. Click here for file

Additional file 4 **All differentially expressed proteins in BR treated sample**. The list was generated by PatternLab. The data from DTASelect were normalized by Row Sigma method and processed by TFold pairwise analysis. The minimum signal in all classes is 2. The cutoff of fold change, p-value and Benjamin-Hochberg (BH) theoretical false-positive rate are 2.0, 0.05 and 0.1, respectively. Click here for file
