# Supplementary material for: Integration of shot-gun proteomics and bioinformatics analysis to explore plant hormone responses
Source: BMC Bioinformatics. 2012 Sep 11;13(Suppl 15):S8. doi: 10.1186/1471-2105-13-S15-S8 (PMC3439724; doi:10.1186/1471-2105-13-S15-S8)
Supplement: Additional file 1 — Workflow of sample preparation and bioinformatics analysis. Additional file 1A shows the workflow of organelle enrichment, protein isolation and 2D LC/MS/MS. Additional file 1B illustrates the flow of protein classification package. Additional file1C shows workflow of traditional plant total protein isolation by TCA/acetone. [file 1471-2105-13-S15-S8-S1.pdf]

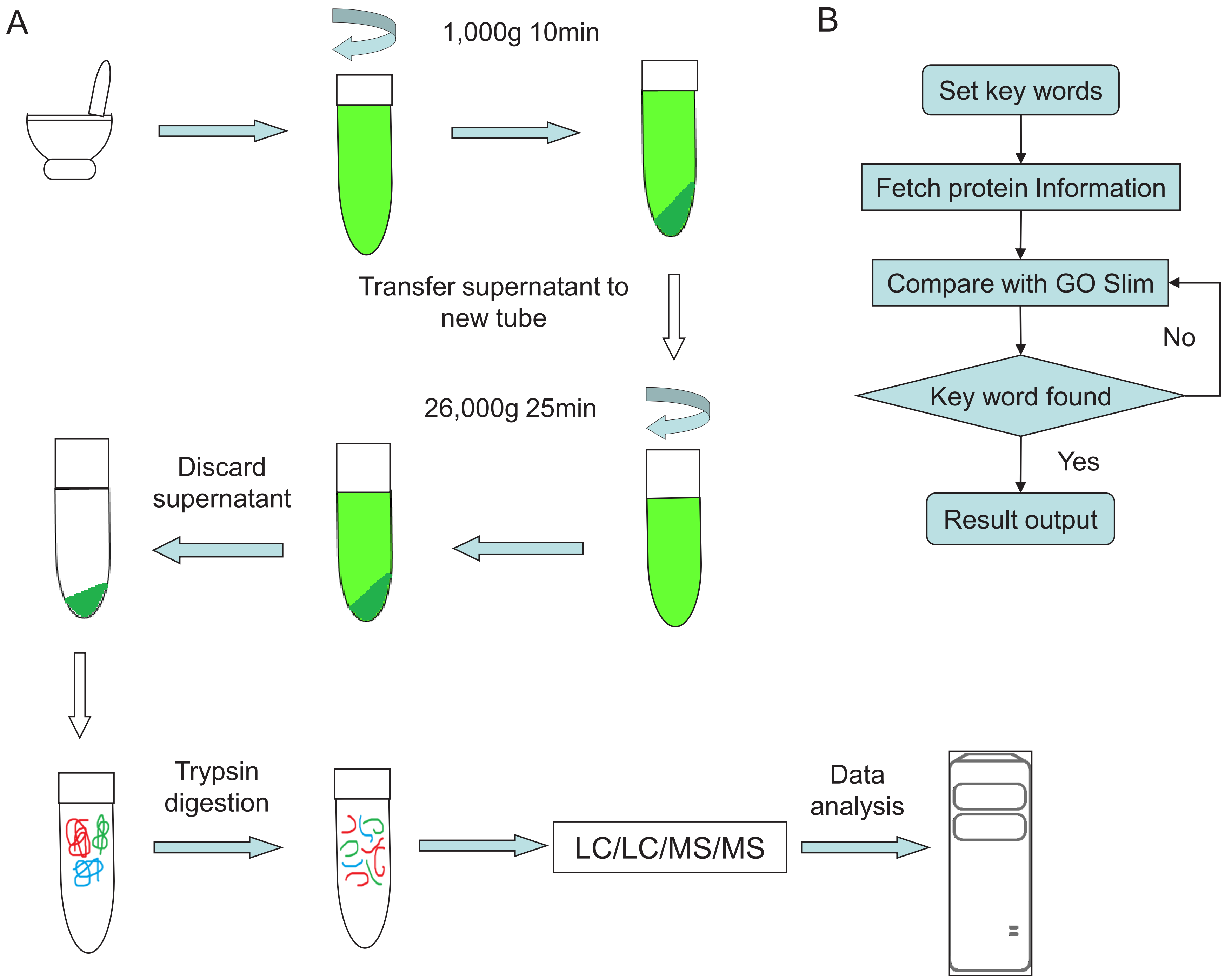
A
1,000g 10min
Transfer supernatant to new tube
26,000g 25min
Discard supernatant
Trypsin digestion
LC/LC/MS/MS
Data analysis
B
Set key words
Fetch protein Information
Compare with GO Slim
No
Key word found
Yes
Result output

C

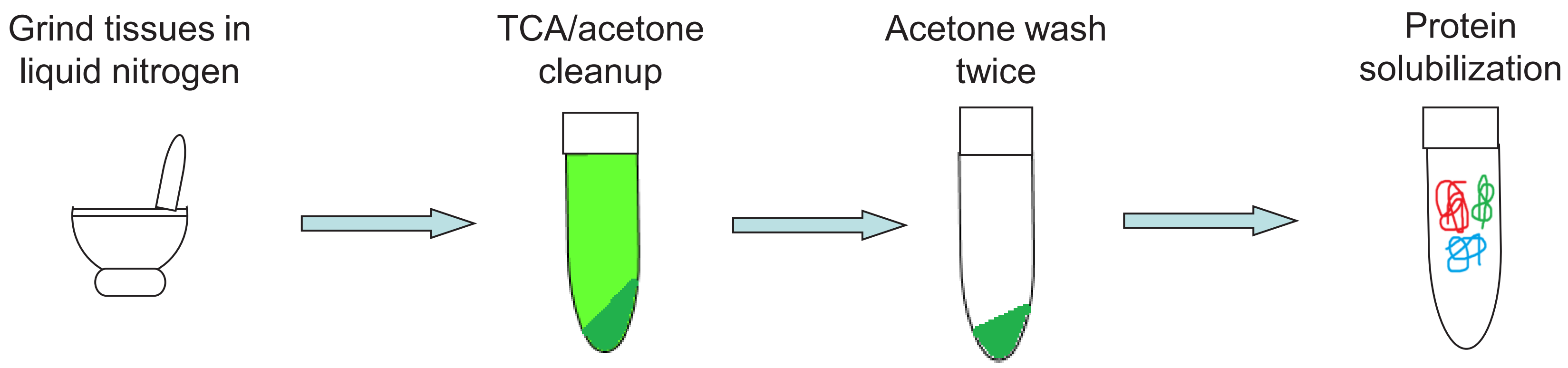

Grind tissues in liquid nitrogen
TCA/acetone cleanup
Acetone wash twice
Protein solubilization
